# Supplementary material for: Reporting Guidelines for Whole-Body Vibration Studies in Humans, Animals and Cell Cultures: A Consensus Statement from an International Group of Experts
Source: Biology (Basel). 2021 Sep 27;10(10):965. doi: 10.3390/biology10100965 (PMC8533415; doi:10.3390/biology10100965)
Supplement: Supplementary file 1 [file biology-10-00965-s001.zip › biology-1381265-supplementary.pdf]

Supplement 1: Outcomes Delphi study for animal and cell cultures research

Table S1. Background characteristics respondents (n=4).

Characteristic	Percentage	M±SD
**Country located (%)**		
United states of America	50	
Canada	50	
**Academic background (%)**		
Biomedical Engineering	25	
Physiology	50	
Biophysical	25	
**Highest academic degree (%)**		
Professor	100	
**Experience with WBV in animals (%)**	100	
**Experience with WBV in cell cultures (%)**	100	
**Experience with WBV research (M±SD, years)**		29.3 ± 3.0
**# of publications WBV animals/cell cultures (M±SD)**		10.0 ± 10.0

Abbreviation: WBV = whole-body vibration.

Table S2. Outcomes Delphi study: information about the vibration. Is it important to report…

	Item	Round 1			Round 2		
		agree	disagree	neutral	agree	disagree	neutral
1	the type of vibration (i.e., synchronous, side-alternating, other)?	100	0	0	100	0	0
2	which frequency (Hz) is used?	100	0	0	100	0	0
3	whether the vibration is immediately full or increases slowly?	50	25	25	75	0	25
4	whether the frequency (Hz) is constant or variable?	100	0	0	100	0	0
5	which peak-to-peak displacement (PPD) / amplitude of the vibration occurs?	100	0	0	75	0	25
6	which peak-acceleration (in multiples of g) occurs?	100	0	0	100	0	0
7	which acceleration (in RMS) occurs?	50	25	25	50	25	25
8	the units of the vibration parameters?	100	0	0	100	0	0
9	the definitions/explanations of the vibration parameters?	75	25	0	100	0	0
10	if manufacturer settings or settings measured by researcher were reported?	100	0	0	100	0	0
11	how the vibration parameters are measured?	100	0	0	100	0	0
12	where on the platform the vibration parameters are measured?	100	0	0	100	0	0
13	how reproducible the vibration parameters are?	75	25	0	100	0	0
14	whether the vibrations are purely sinusoidal?	100	0	0	100	0	0
15[a]	if unidirectional vibrations are vertical or horizontal				100	0	0
16[a]	if "how much vertical vs horizontal vibrations" is measured by 3D accelerometry?				100	0	0
17[a]	if accelerometers are ATTACHED to the cell culture plate?				100	0	0
18[a]	the duration of vibration, number of bouts and intervals between bouts?				100	0	0

[a]Added on round 2 based on the suggestions of the respondents in round 1.

Table S3. Outcomes Delphi study: information about the device. Is it important to report…

	Item	Round 1			Round 2		
		agree	disagree	neutral	agree	disagree	neutral
1	the manufacturer, device specifications, and production type?	75	25	0	100	0	0
2	the size of the vibration platform?	25	50	25	75	25	0
3	whether changes are made to the device (e.g., mounting a cage on it)?	100	0	0	100	0	0
4	the dimensions of the cage or container in which the animal(s) was (were) placed (if any) during WBV?	75	0	25	75	0	25
5	how the energy is generated (e.g., direct mechanical transmission or electromagnetic transmission) as this can have an effect on the performance of the device?	50	25	25	50	0	50
6[a]	if animals were on all 4 paws or were able to (or did) rear up during vibration exposure?				100	0	0
7[a]	how closely the sham conditions mimic those encountered by the animals treated with vibration?				100	0	0

[a]Added on round 2 based on the suggestions of the respondents in round 1.

Table S4. Outcomes Delphi study: information about the administration. Is it important to report…

	Item	Round 1			Round 2		
		agree	disagree	neutral	agree	disagree	neutral
1	the number of sessions (per day or week) with WBV?	100	0	0	100	0	0
2	the number of exposures to WBV within one session?	100	0	0	100	0	0
3	the time between sessions of WBV?						
4	the on vs. off-times of vibration (e.g., pauses and how long) within one session?	100	0	0	100	0	0
5	the total exposure time to WBV across all sessions?	75	0	25	75	0	25
6	the conditions of the test room (e.g., light and temperature)?	50	50	0	100	0	0
7	the location of the intervention (e.g., a separated experimental room or the room where the subjects are housed during the experiment)?	50	25	25	75	0	25
8	whether (motor) behavior is being monitored and/or reduced during the WBV session?	75	25	0	100	0	0
9	which posture or body position the subjects take on during the vibration (e.g., sitting, standing, lying, etc.) depending on the species used?	100	0	0	100	0	0
10	whether the position/posture changes during WBV?	100	0	0	100	0	0
11	whether subjects were restrained or not during WBV?	100	0	0	100	0	0
12	the time of day the WBV session takes place (in relation to the L/D cycle)?	50	50	0	100	0	0
13	the subjects' body weight before and after the entire WBV protocol?	75	0	25	100	0	0
14	whether the subjects were habituated to the device before the start of the WBV protocol?	75	0	25	75	0	25
15	species-specific inclusion/exclusion criteria?	100	0	0	100	0	0
16	whether whiskers were present/intact (when species are used having whiskers)?	0	50	50	75	0	25
17	whether the animals were anesthetized or not during the WBV session?	100	0	0	100	0	0
18	whether the animals were housed individually or in groups	100	0	0	100	0	0

	during a WBV session on the vibrating plate?			
19[a]	to what conditions EXACTLY the normal control animals/cell cultures were subjected to, in comparison to vibrated experimental animals/cell cultures?	100	0	0

[a]Added on round 2 based on the suggestions of the respondents in round 1.
